# A Novel Low Temperature PCR Assured High-Fidelity DNA Amplification

**DOI:** 10.3390/ijms140612853

**Published:** 2013-06-20

**Authors:** Guofan Hong, Sin Hang Lee, Shichao Ge, Shaoxia Zhou

**Affiliations:** 1 State Key Laboratory of Molecular Biology, Institute of Biochemistry and Cell Biology, Shanghai Institutes for Biological Sciences, Chinese Academy of Sciences, 320 Yueyang Road, Shanghai 200031, China; E-Mails: scge@sibcb.ac.cn (S.G.); sxzhou@sibcb.ac.cn (S.Z.); 2 Milford Hospital and Milford Molecular Laboratory, 2044 Bridgeport Avenue, Milford, CT 06460, USA; E-Mail: shlee01@snet.net

**Keywords:** low temperature PCR, high-fidelity DNA amplification, LoTemp PCR, high processivity

## Abstract

As previously reported, a novel low temperature (LoTemp) polymerase chain reaction (PCR) catalyzed by a moderately heat-resistant (MHR) DNA polymerase with a chemical-assisted denaturation temperature set at 85 °C instead of the conventional 94–96 °C can achieve high-fidelity DNA amplification of a target DNA, even after up to 120 PCR thermal cycles. Furthermore, such accurate amplification is not achievable with conventional PCR. Now, using a well-recognized L1 gene segment of the human papillomavirus (HPV) type 52 (HPV-52) as the template for experiments, we demonstrate that the LoTemp high-fidelity DNA amplification is attributed to an unusually high processivity and stability of the MHR DNA polymerase whose high fidelity in template-directed DNA synthesis is independent of non-existent 3′–5′ exonuclease activity. Further studies and understanding of the characteristics of the LoTemp PCR technology may facilitate implementation of DNA sequencing-based diagnostics at the point of care in community hospital laboratories.

## 1. Introduction

Polymerase chain reaction (PCR) is commonly used to generate specific primer-defined amplicons, usually catalyzed by a thermophilic DNA polymerase and carried out in a thermal cycler programmed for DNA denaturation at 94–96 °C, primer annealing at 53–67 °C and primer extension at 72 °C. Pre-PCR purification of DNA in complex human samples is usually required, but may risk losing the target DNA altogether when the latter is low in quantity. Recently, re-amplification of the target DNA amplicon by a moderately heat-resistant (MHR) DNA polymerase, which is conducted in a low temperature (LoTemp) PCR thermal cycling program set for DNA denaturation at 85 °C, primer annealing at 40–50 °C and primer extension at 65 °C, has been used to prepare templates for DNA sequencing-based molecular diagnosis of infection caused by *Borrelia burgdorferi* [1,2], human papilloma virus (HPV) [3,4], *Neisseria gonorrhoeae* [5,6] and *Chlamydia trachomatis* [5,6]. High-fidelity re-amplification of target DNA amplicons in a nested PCR setting can overcome the effects of competitive inhibitors, which usually require pre-PCR purification to remove them from complex clinical samples [4]. A same-nested LoTemp PCR repeated for 120 thermal cycles has successfully re-amplified the amplicon of an HPV-16 L1 gene DNA in postmortem materials to prepare the template for direct DNA sequencing after the conventional PCR failed to do so [7]. LoTemp nested PCR has generated specific amplicons from the HPV L1 gene DNA fragments bound to an insoluble aluminum salt adjuvant [8,9], while *Taq* nested PCR yields a mixture of specific and non-specific products [9].

High-fidelity PCR re-amplification of an amplicon is difficult to achieve with thermophilic DNA polymerases, due to enzyme-induced mutations and chimeric sequences as the number of PCR cycles increases [10–15]. In this article, we report an unusually high processivity and stability of a MHR DNA polymerase, which may account for the high-fidelity amplification of LoTemp PCR. We selected a well-recognized primary PCR amplicon of the HPV-52 L1 gene and its heminested PCR amplicons as the sample templates to demonstrate some of the characteristics of this LoTemp PCR technology.

## 2. Results and Discussion

### 2.1. LoTemp PCR Extension of Primer with Mismatched Base at the 3′-Terminus

**Figure 1:**
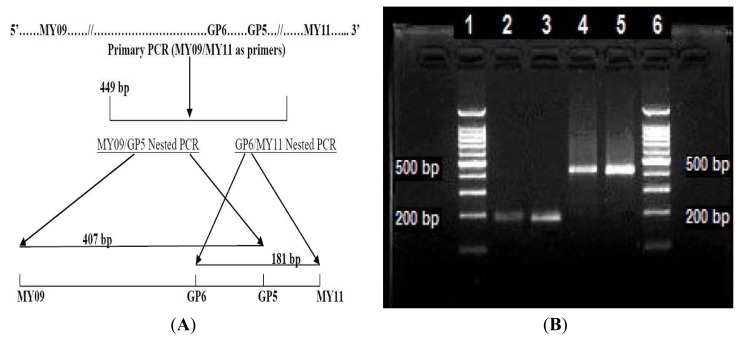
Hypervariable segment of the HPV-52 L1 gene selected for the study. (**A**) Schematic position map of the consensus primer binding sites in the HPV-52 L1 gene region (Locus GQ472848); (**B**) Agarose gel electrophoresis plate showing the GP6/MY11 and GP5/MY09 heminested PCR amplicons of the hypervariable L1 gene region of a wild-type HPV-52 isolate from the cervicovaginal cells of a patient, generated by a MHR DNA polymerase.

The schematic position map of the consensus primer binding sites in the HPV-52 L1 gene region (Locus #GQ472848.1) is illustrated in Figure 1A. The base sequences of the PCR primers are MY09 = 5′-CGTCCMARRGGAWACTGATC-3′ and MY11 = 5′-GCMCAGGGWCATAAY AATGG-3′ [16] and GP5 = 5′-TTTGTTACTGTGGTAGATAC-3′ and GP6 = 5′-GAAAAATAAA CTGTAAATCA-3′ [17]. Key to degeneracy is: M = (A + C), R = (A + G), W = (A + T), Y = (C + T). After agarose gel electrophoresis, the GP6/MY11 and GP5/MY09 heminested PCR amplicons generated by the MHR DNA polymerase are shown in Figure 1B. Lanes 1 and 6 = DNA rulers; Lanes 2 and 3 = 181 bp GP6/MY11 heminested PCR amplicon band in duplicate; Lanes 4 and 5 = 407 bp GP5/MY09 heminested PCR amplicon band in duplicate.

**Figure 2:**
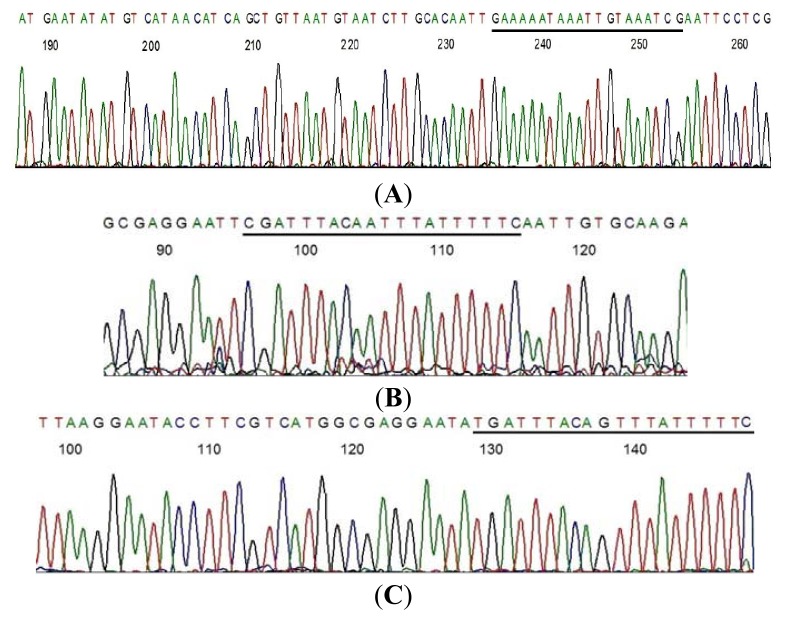
DNA sequencing showing extension of primer with a 3′-terminal mismatch. (**A**) Computer-generated electropherogram showing the sequence of a wild-type HPV-52 L1 gene fragment; (**B**) A short sequence of a wild-type HPV-52 L1 gene, using GP5 as the sequencing primer, confirming that the GP6-binding site sequence is 5′-CGATTTACAATTTATTTTTC-3′; (**C**) Electropherogram showing the sequence of the GP6 binding site of a GP6/MY11 heminested PCR amplicon to be 5′-TGATTTACAGTTTATTTTTC-3′ (underlined), using MY11 as the sequencing primer.

The MY09 primer and the GP5 primer were used as the sequencing primers to perform two-directional DNA sequencing of the GP5/MY09 heminested PCR amplicon to confirm that the DNA sequence of the GP6 primer-binding site in this wild HPV-52 DNA was 5′-CGATTTACAATTTATTTTTC-3′ (Figure 2A,B), as recorded in the GenBank (Locus #GQ472848.1). Then a GP6/MY11 heminested PCR amplicon was sequenced, using the MY11 PCR primer as the sequencing primer. The latter sequencing electropherogram showed that the GP6/MY11 heminested PCR amplicon contained a sequence of 5′-TGATTTACAGTTTATTTTTC-3′ at its GP6 binding site (Figure 2C), with no base substitution for the A/C mismatch at the 3′-terminus of the incorporated consensus GP6 primer whose sequence is 5′-GAAAAATAAACTGTAAATCA-3′.

The above sequencing electropherograms showed that the LoTemp PCR is able to initiate its high-fidelity DNA synthesis without a correction of the A/C mismatch at the 3′-terminus of the GP6 primer, which is highly unusual because PCR primers with a 3′-terminal “A” mismatch are known to be less efficiently amplified regardless of the corresponding nucleotide on the template strand [18]. In addition, there was an A/A mismatched incorporation immediately after the primer, indicating that a topological condition created by the mismatch at the 3′-terminus of the primer prevented the DNA polymerase from performing its proofreading function at the very first base synthesis. The results challenge the general assumption that specificity and fidelity of DNA polymerases depend on their ability to bind and extend matched, but not mismatched, nucleotides at the 3′ end of a primer annealed to a complementary template strand [13–15,18].

### 2.2. High Amplicon Yield by LoTemp PCR

**Figure 3:**
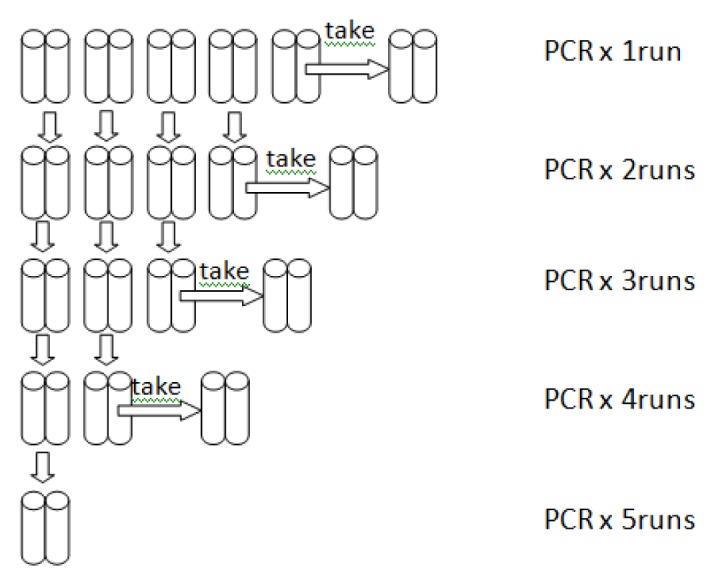
Design to study the kinetics of amplicon generation in *Taq* PCR and LoTemp PCR.

To explore the kinetics of amplicon generation by the MHR polymerase and by a thermophilic *Taq* DNA polymerase, we set up 10 MHR polymerase nested PCRs and 10 *Taq* nested PCRs, each containing 1 μL template, 1 μL MY09 primer, 1 μL GP5 primer, 1 μL 5 mM dNTPs and one unit of DNA polymerase in a total volume of 25 μL with MgCl_2_. The MHR polymerase PCR tubes were subjected to a series of LoTemp PCR 30-cycle runs and the *Taq* PCR tubes to a series of conventional PCR 30-cycle runs, so that two PCR tubes were removed from each of the two thermal cyclers after completion of each 30-cycle run until all the PCR tubes in the cyclers were exhausted, as schematically illustrated in Figure 3.

**Figure 4:**
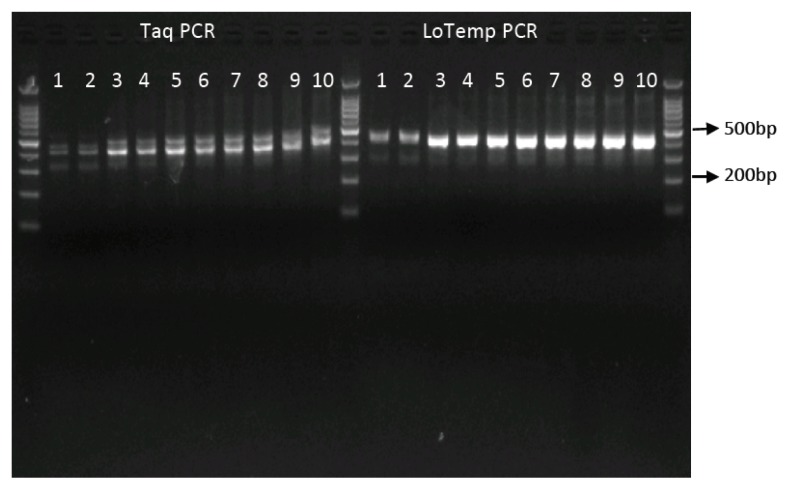
Products generated by a *Taq* and a MRH polymerase LoTemp PCR after 1–5 PCR runs. Lanes (1,2), (3,4), (5,6), (7,8) and (9,10) represent identical PCR × 1, 2, 3, 4 and 5 runs, respectively, in duplicate. *Taq* PCR with a *TaKaRa Taq* polymerase; LoTemp PCR with a MRH polymerase.

A 5 μL aliquot of PCR products from each of the PCR tubes was mixed with ethidium bromide and quantified after electrophoresis in the agarose gel, as shown in Figure 4.

**Figure 5:**
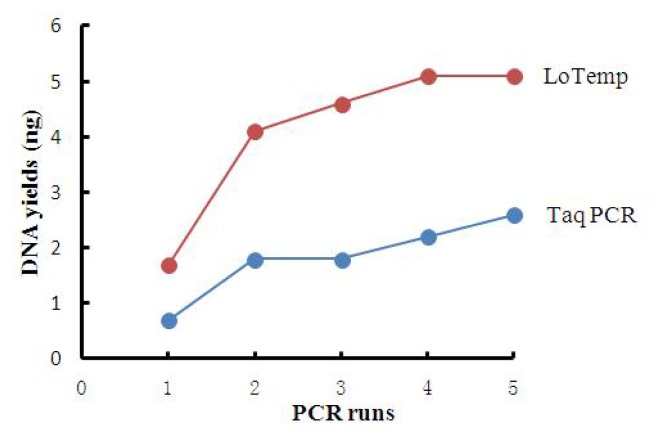
DNA product yields (ng) by *Taq* PCR and LoTemp PCR expressed graphically.

The average yields of PCR products generated by the LoTemp PCR were more than double those generated by *Taq* PCR after any number of runs, as summarized in Table 1 and Figure 5.

The results indicate a much higher processivity and stability of the MHR DNA polymerase, compared to those exhibited by a *Taq* enzyme during multiple consecutive PCR runs. The conventional *Taq* amplification also generated a non-specific amplicon band in the nested PCR products visible in all lanes (Figure 4, *Taq* PCR).

### 2.3. High Processivity of LoTemp PCR

**Figure 6:**
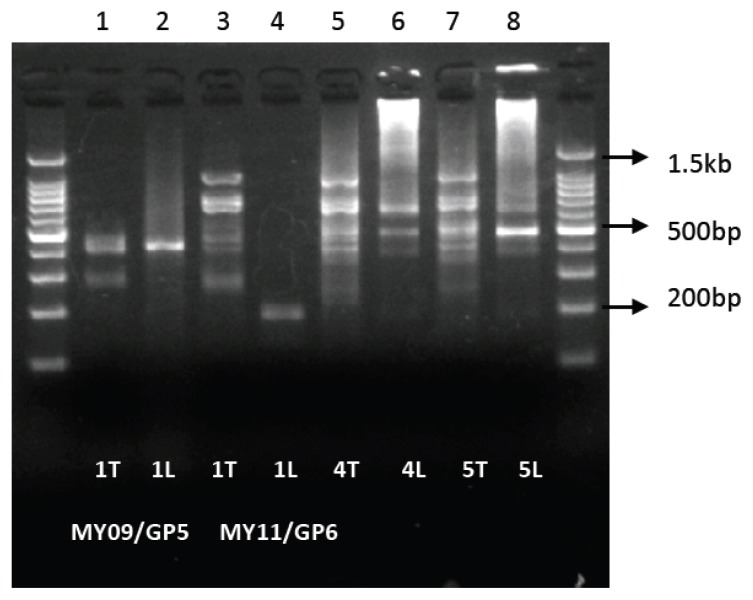
Processivity of LoTemp PCR (Lanes 2,4,6,8) *vs. Taq* PCR (Lanes 1,3,5,7). Eight PCR tubes, numbered 1 to 8, were prepared, each containing 12 ng DNA from an HPV-52 MY09/MY11 primary PCR amplicon in 1 μL as the heminested PCR template. To tubes 1, 3, 5 and 7 were added 22 μL of conventional PCR ingredients with one unit of *Taq* DNA polymerase, complete minus the primers. To tubes 2, 4, 6 and 8 were added 22 μL of all LoTemp PCR ingredients with one unit of MHR DNA polymerase, complete minus the primers. To complete the total 25 μL PCR volume, 1 μL of GP5 primer and 1 μL of MY09 primer were added to tubes #1 and #2; and 1 μL of GP6 primer and 1 μL of MY11 primer were added to all remaining tubes, #3 through #8. Then the eight PCR tubes were subjected to different thermal cycling programs as follows: Tube 1(1T): Primers GP5/MY09, conventional *Taq* PCR 30 thermal cycles ×1 run; Tube 2(1L): Primers GP5/MY09, LoTemp PCR 30 thermal cycles ×1 run; Tube 3(1T): Primers GP6/MY11, conventional *Taq* PCR 30 thermal cycles ×1 run; Tube 4(1L): Primers GP6/MY11, LoTemp PCR 30 thermal cycles ×1 run; Tube 5(4T): Primers GP6/MY11, conventional *Taq* PCR 30 thermal cycles ×4 runs; Tube 6(4L): Primers GP6/MY11, LoTemp PCR 30 thermal cycles ×4 runs; Tube 7(5T): Primers GP6/MY11, conventional *Taq* PCR 30 thermal cycles ×5 runs; Tube 8(5L): Primers GP6/MY11, LoTemp PCR 30 thermal cycles ×5 runs. The image of the products generated in the eight PCR tubes as analyzed by agarose gel electrophoresis is presented here in the corresponding gel Lanes 1–8. The images in Lanes 1 and 2 confirmed that the LoTemp nested PCR (Lane 2) generated a more specific 407 bp GP5/MY09 amplicon than the conventional *Taq* nested PCR (Lane 1) after one PCR run. The LoTemp nested PCR (Lane 4), and not the conventional *Taq* nested PCR (Lane 3), yielded a specific 181 bp HPV DNA amplicon terminated by a pair of GP6/MY11 primers after one PCR run. After four or five PCR runs, the LoTemp nested PCR generated a high molecular-weight amplicon of about 8,000 bp in size, consistent with the full circular size of the HPV-52 whole genome (Lanes 6 and 8), using traces of the original HPV-52 DNA as the template. The corresponding conventional *Taq* nested PCR failed to produce such high molecular weight amplicons (Lanes 5 and 7).

For another comparative processivity study, a series of 25 μL LoTemp heminested PCRs and 25 μL *Taq* heminested PCRs were set up as described in section 2.2, except that the GP6/MY11 PCR primer pair was also used along with the GP5/MY09 pair in parallel. The PCR tubes were subjected to one, four and five runs of 30 LoTemp thermal cycles for the MHR polymerase, and one, four and five runs of 30 conventional thermal cycles for the *Taq* enzyme, respectively. The products generated in the PCR tubes as analyzed by electrophoresis showed that a single run of a 30-cycle LoTemp PCR yielded a narrow band of 407 bp GP5/MY09 target DNA heminested PCR amplicon (Figure 6, Lane 2), whereas the corresponding conventional *Taq* PCR generated a poorly defined diffuse band (Figure 6, Lane 1). When the GP6/MY11 primer pair was used, a single 30-cycle LoTemp heminested PCR run generated a distinct 181 bp target DNA amplicon (Figure 6, Lane 4). The *Taq* polymerase generated no visible GP6/MY11 amplicon, but numerous non-specific PCR products after a single PCR run (Figure 6, Lane 3), due to its failure to extend the GP6 primer. When there is a lack of specific duplex in the PCR mixture, a DNA polymerase may cause non-specific DNA polymerization at random if other DNA fragments with sequences partially complementary to the primers are present. However, such non-specific DNA amplification was readily suppressed when the same samples were spiked with a specific template [1]. In the current example, we show that a highly processive DNA polymerase capable of extending an otherwise well matched primer with a 3′-terminal mismatch also avoids non-specific PCR amplification (Figure 6, Lanes 3 and 4).

In the PCRs where the GP6/MY11 primer pair was used and the number of PCRs increased to four and five runs (Figure 6, Lanes 6 and 8), the initial 181 bp HPV amplicon generated in the LoTemp PCR vanished from the agarose gel. Instead, an around 8,000 bp amplicon appeared at the very top of the gel (Figure 6, Lanes 6 and 8), indicating that the 181 bp dsDNA had become ssDNA at the temperature of denaturation, and the 181-base ssDNA had served as the primers for the generation of a large amplicon of the circular HPV-52 genome (7,960 bp) after the initial GP6 and MY11 primers were exhausted. Traces of the whole HPV genomic DNA are invariably carried over from the original samples to the nested PCR when inter-step purification is not carried out. In the corresponding conventional *Taq* PCR, no specific GP6/MY11 PCR amplicons were visible (Figure 6, Lanes 3, 5, 7). In a previously reported parallel comparative study, the conventional *Taq* PCR failed to yield an HPV DNA amplicon in four of seven (4/7) clinical cervicovaginal specimens, which were infected by HPV proven by LoTemp nested PCR followed by direct DNA sequencing [4].

## 3. Experimental Section

### 3.1. Preparation of the 449 bp HPV-52 L1 Gene MY09/MY11 Primary PCR Amplicon

A 0.5 mL aliquot of a cervicovaginal cell suspension, which was collected from a patient infected with HPV-52 and preserved in Surepath^®^ fixatives (BD Diagnostics-TriPath, Burlington, NC, USA) was centrifuged in a 1.5 mL tube at 16,000× *g* for 5 min in an Eppendorf microcentrifuge (model 5424) equipped with a corresponding rotor (model FA45-24-11). The fixed cells were washed in 1 mL reagent grade water and then in 1 mL of washing buffer consisting of 50mM Tris-HCl, 1 mM EDTA, 0.5% Tween 20, pH 8.1. The washed cell pellet was re-suspended and digested at 45–55 °C overnight in 100 μL of 0.1 mg/mL proteinase K (Sigma Chemical Co., St. Louis, MO, USA) dissolved in the same washing buffer. After denaturing the proteinase in the cell digestate in a metal block at 95 °C for 10 min and after a final centrifugation of the digestate at 16,000× *g* for 5 min, the supernatant, referred to as the proteinase K digestate, was used as the source of the initial template to be amplified by a pair of MY09 and MY11 degenerate primers with LoTemp PCR. The primary PCR mixture contained 1 μL of the proteinase K digestate, 1 μL of 10 μM MY09 primer, 1 μL of 10 μM MY11 primer, 2 μL of ddH_2_O and 20 μL of LoTemp^®^ ready-to-use master mix (Cat. No. #8802, HiFi DNA Tech, LLC, Trumbull, CT, USA) with all the components needed for low temperature PCR, including one unit of a moderately heat-resistant HiFi^®^ DNA polymerase, 1 μL of 5 mM dNTPs and optimized Mg^2+^, buffer, proprietary dsDNA-melting agents and preservatives, to reach a total 25 μL reaction volume. For thermocycling, the temperature steps of a TC-412 Thermal Cycler (Techne Incorporated, Burlington, NJ, USA) were programmed for an initial heating at 85 °C for 10 min, followed by 30 cycles at 85 °C for 30 s, 40 °C for 30 s and 65 °C for 1 min. The final extension was 65 °C for 10 min. The MY09/MY11 primary PCR products were adjusted to a concentration containing 12 ng of the 449 bp amplicon per 1 μL, which was used as the template for various heminested PCRs as presented in the main text.

### 3.2. LoTemp Heminested PCR Amplicon as Template for DNA Sequencing

One microliter containing 12 ng of the 449 bp MY09/MY11 primary PCR amplicon was transferred into a 24 μL LoTemp heminested PCR mixture, consisting of 20 μL of LoTemp^®^ HiFi^®^ DNA polymerase ready-to-use master mix (Cat. No. #8802, HiFi DNA Tech, LLC, Trumbull, CT, USA), 2 μL of ddH_2_O, 1 μL of 10 μM MY11 (or MY09) primer and 1μL of 10 μM GP6 (or GP5) primer. The PCR mixture was subjected to a 30-cycle of LoTemp amplification, as described for the primary PCR to generate a GP6/MY11 (or a GP5/MY09) heminested PCR amplicon for direct DNA sequencing.

### 3.3. Direct DNA Sequencing

One microliter of the HPV-52 GP5/MY09 or GP6/MY11 heminested PCR amplicon without purification was used as the template for the fluorescent dye terminator reaction. The sequencing primer was 1 μL of 10 μM of the MY09, GP5 or MY11 solution. In addition to the template and primer, the modified Sanger reaction mixture contained 1 μL of the BigDye^®^ Terminator (v 1.1/Sequencing Standard Kit, Foster City, CA, USA), 3.5 μL 5× buffer, and 13.5 μL ddH_2_O in a total volume of 20 μL for 20 enzymatic primer extension/termination reaction cycles according to the protocol supplied by the manufacturer (Applied Biosystems, Foster City, CA, USA). As recommended by the manufacturer, the cycling steps consisted of an initial heating at 96 °C for 4 min, followed by 96 °C for 10 s, 50 °C for 5 s and 60 °C for 4 min per cycle for a total of 20 cycles, with the final hold at 4 °C. After dye-terminator cleanup with a Centri-Sep column (Princeton Separations, Adelphia, NJ, USA), the reaction mixture was loaded in an automated ABI 3130 four-capillary Genetic Analyzer for sequence analysis. Representative segments of the computer-generated base-calling electropherograms are illustrated in Figure 2.

## 4. Conclusions

Non-heat-resistant polymerases with high processivity and high fidelity in DNA synthesis without a companion 3′–5′ exonuclease activity, such as the modified T7 [19] and the Bst [20] DNA polymerases, have been used for DNA sequencing. These enzymes cannot be used for PCR because they are unable to survive the high temperature generally required for dsDNA denaturation. DNA polymerases with high processivity are usually associated with high fidelity in template-directed DNA polymerization [21]. Enhancing the processivity of thermophilic DNA polymerases has been an ongoing quest for many researchers with little success [22]. Certain DNA polymerases of moderate heat resistance may have higher fidelity in template-directed DNA synthesis than the conventional thermophilic DNA polymerases. When used in LoTemp PCR in which denaturation of dsDNA is accomplished by chemical-assisted thermal melting with reduced heat, a suitable MHR DNA polymerase may be useful in performing routine nested PCR to facilitate implementation of DNA sequencing-based diagnostic tests in community hospital laboratories.

## Figures and Tables

**Table 1:** DNA product yields (ng, the average of duplicate) by *Taq* PCR (T) and LoTemp PCR (L) after 1–5 PCR runs.

PCR runs	1	2	3	4	5
DNA yields by T	0.7	1.8	1.8	2.2	2.6
DNA yields by L	1.7	4.1	4.6	5.1	5.1
